# Necrosis of the Ventral Penile Skin Flap: A Complication of Hypospadias Surgery in Children

**DOI:** 10.1155/2015/452870

**Published:** 2015-04-01

**Authors:** Ünal Bakal, Musa Abeş, Mehmet Sarac

**Affiliations:** ^1^Department of Pediatric Surgery, Faculty of Medicine, Firat University, 23119 Elazig, Turkey; ^2^Department of Pediatric Surgery, Faculty of Medicine, Adiyaman University, 23119 Elazig, Turkey

## Abstract

*Objectives*. To review cases of hypospadias that were repaired with TIPU method and consequently resulted in the necrosis of ventral penile skin flaps. *Methods*. Eighty-three patients with hypospadias underwent TIPU procedure by two surgeons. Neourethra in all patients was covered with dartos flap prepared from the preputium or penile shaft. In cases where ventral skin could not be covered primarily, closure was ensured by using preputial Ombredanne or Byars' flaps to repair ventral defects. *Results*. The median age of patients was 4 years. Twenty-five (30.12%) patients that underwent hypospadias repair had urethral opening at the coronal level, 33 (39.75%) at the distal penis, 10 (12.04%) at the midpenis, and 15 (18.07%) at the proximal penis. The ventral skin defect could not be primarily covered in 10 patients with penile shaft hypospadias. Consequently, Byars' method was used in 8 of these patients to cover the defect and the Ombredanne method was used in the remaining 2. Ventral skin flap necrosis developed in 5 patients (4 Byars and 1 Ombredanne). It was medically treated in 4 patients. Urethral fistula developed in the other patient whose necrosis was deeper. The mean hospital stay was 7 days for patients without necrosis, and 14 for those with necrosis. *Conclusion*. We are of the opinion that dartos flaps used in the TIPU method in order to cover neourethra and decrease the incidence of fistula development lead to necrosis in the Ombredanne or Byars' flaps by causing low blood supply to the preputium and thus extend hospital stay.

## 1. Introduction

In the tubularized incised plate urethroplasty (TIPU) method which uses urethral plates for urethroplasty and the Mathieu procedure which uses proximal parameatal skin flaps, it is sometimes not possible to primarily cover the skin on the ventral surface of the penis after chordee release and urethroplasty. For this purpose, the Ombredanne or Byars' method may be used to transfer preputial skin to the ventral side [1, 2]. In this study, we retrospectively reviewed cases of hypospadias that were repaired with the TIPU method and resulted in the necrosis of ventral penile skin flaps.

## 2. Material and Method

In our clinic, a total of 111 children received hypospadias repair with different techniques performed by 2 different surgeons between January 2008 and December 2011. We studied 83 of these cases that were repaired with the TIPU method. In all patients, penile skin was degloved to the penoscrotal junction. The dartos flap big enough to fully cover the neourethra was prepared from preputial or penile shaft. Dorsal plication was performed in patients with persisting chordee. Prior to urethroplasty and glanuloplasty, prepitium and penile skin was excluded and a tourniquet was placed on the radix penis. Urethroplasty was performed with 7/0 or 6/0 continuous subcutaneous, and glanuloplasty was performed with 6/0 interrupted polyglactin or polydioxanone sutures. The neourethra was covered with the previously prepared dartos flap. Drainage was done with a nelaton catheter size 6 or 8. It was withdrawn on day 7 on average. In cases where ventral skin could not be covered primarily, closure was ensured by using preputial Ombredanne or Byars' flaps to repair ventral defects. The skin was covered with 6/0 or 7/0 interrupted polyglactin sutures. The dressing was removed between days 3 and 5. Antibiotics were used in all cases (cefazoline 100 mg/kg/day/3 doses) and oxybutynin was used in some cases (2 mg/kg/2 doses). A rifampicin dressing was used for signs of necrosis in ventral skin.

## 3. Results

**Figure 1:**
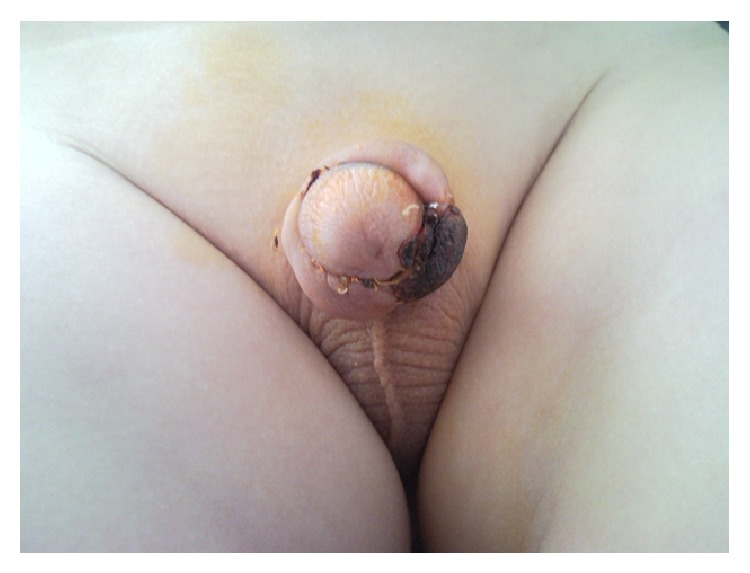
Patient in whom ventral penile skin defect was covered with Byars' flap and superficial necrosis developed.

**Figure 2:**
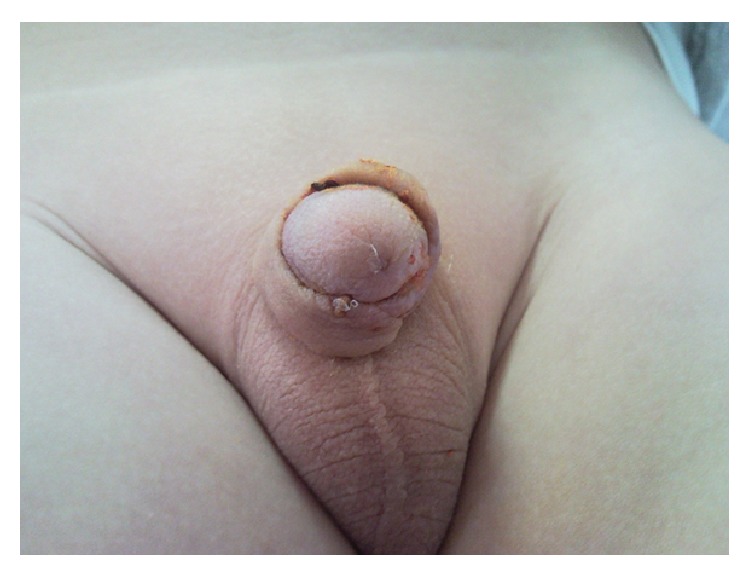
Same patient after recovery following medical treatment.

The median age of patients was 4 years (ranging between 1 and 14 years). Twenty-five (30.12%) patients that underwent hypospadias repair had urethral opening at the coronal level, 33 (39.75%) at the distal penis, 10 (12.04%) at the midpenis, and 15 (18.07%) at the proximal penis. The ventral skin defect could not be primarily covered in 10 patients with penile shaft hypospadias. Consequently, Byars' method was used in 8 of these to cover the defect and the Ombredanne method in the remaining 2. Necrosis developed in the skin flap of 5 cases (50%) (4 Byars and 1 Ombredanne). It was superficial and medically treated in 4 (Figures 1 and 2). Urethral fistula developed in the other patient whose necrosis was deeper. The mean hospital stay was 7 days for patients without necrosis and 14 for those with necrosis (between 13 and 15 days).

## 4. Comment

The tubularized incised plate urethroplasty method is currently the most popular hypospadias treatment worldwide [3]. It may be used to repair all of distal and most of proximal hypospadias cases [3–5]. It may be compared to previous techniques with regard to complications, but cosmetically it is superior to them [6]. Despite their low incidence rate when compared to other techniques, the most commonly reported complications of the TIPU procedure include formation of fistula 0–39% (mean 5%) and meatal stenosis 0–32% (mean 3%) [3, 6, 7]. In our series, the most common complications were ventral penile skin flap necrosis (6%) and urethral fistula (1,2%).

The causes of skin flap necrosis listed in the literature are hematoma, infections, vascular spasms, and tight dressings [7]. Hematoma and infection were not developed in any of our cases. Tightness of the dressing was equal for all the cases. We are of the opinion that this problem may be related to the dartos flap as skin necrosis was only limited to those that received ventral skin flap and then only to the flap region among our patients, and no other factors existed that might cause necrosis.

In the TIPU procedure, dartos fascia freed from the preputium and the dorsal side of the penis and/or the ventral shaft is used to fully cover the neourethra in order to prevent urethral fistula despite the debated effectiveness of this method [4, 8, 9]. We believe that aggressively freeing the dartos from the preputium and the penile shaft leads to vascular failure in flaps prepared from preputium and dorsal skin, and consequently to necrosis. We attribute the low incidence of fistula formation in our series and the high incidence of ventral skin flap necrosis to the dartos flap. We believe that the dartos used to cover the neourethra prevents fistula but causes necrosis in the preputial ventral skin flaps. Necrosis was superficial in 4 of the 5 cases, who were treated with conservative therapy. Full necrosis and fistula developed only in one patient who recovered in the late stage. We are of the opinion that patients may be followed up with conservative therapy during the early stages for as long as necrosis does not extend, and surgery should be avoided.

## 5. Conclusion

Particularly in cases where the ventral surface of the penis cannot be covered primarily and preputial skin flaps may be necessary to cover the defect, the dartos flap should not be used aggressively to cover the neourethra; the ventral dartos flap should be used whenever possible instead of preputial and dorsal penile shaft dartos flap; and possible low blood supply to preputial skin flap and ultimately necrosis should be prevented.
